# Priorities and realities: addressing the rich-poor gaps in health status and service access in Indonesia

**DOI:** 10.1186/1475-9276-10-47

**Published:** 2011-11-09

**Authors:** Budi Utomo, Purwa K Sucahya, Fita R Utami

**Affiliations:** 1 Department of Biostatistics and Population, Faculty of Public Health University of Indonesia, Depok, Indonesia; 2 Center for Health Research, Faculty of Public Health University of Indonesia, Depok, Indonesia

**Keywords:** health inequity, child and infant mortality, prevalence of underweight children, health service access, institutional and policy factors, Indonesia

## Abstract

**Introduction:**

Over the past four decades, the Indonesian health care system has greatly expanded and the health of Indonesian people has improved although the rich-poor gap in health status and service access remains an issue. The government has been trying to address these gaps and intensify efforts to improve the health of the poor following the economic crisis in 1998.

**Methods:**

This paper examines trends and levels in socio-economic inequity of health and identifies critical factors constraining efforts to improve the health of the poor. Quantitative data were taken from the Indonesian Demographic Health Surveys and the National Socio-Economic Surveys, and qualitative data were obtained from interviews with individuals and groups representing relevant stakeholders.

**Results:**

The health of the population has improved as indicated by child mortality decline and the increase in community access to health services. However, the continuing prevalence of malnourished children and the persisting socio-economic inequity of health suggest that efforts to improve the health of the poor have not yet been effective. Factors identified at institution and policy levels that have constrained improvements in health care access and outcomes for the poor include: the high cost of electing formal governance leaders; confused leadership roles in the health sector; lack of health inequity indicators; the generally weak capacity in the health care system, especially in planning and budgeting; and the leakage and limited coverage of programs for the poor.

**Conclusions:**

Despite the government's efforts to improve the health of the poor, the rich-poor gap in health status and service access continues. Factors at institutional and policy levels are critical in contributing to the lack of efficiency and effectiveness for health programs that address the poor.

## Introduction

As mandated by the National Constitution, the government has put efforts into providing quality health services to all. Over the past four decades, the Indonesian health care system has greatly expanded. Public and private hospitals are now available at district levels. Half of the hospitals are privately run; and most doctors have dual practices in the public and private sectors [1]. Nevertheless, the number of hospital beds per capita is lower in comparison to other similar income countries. The available beds are poorly utilized with an average occupancy rate of 60 percent [2]. The reasons for low availability of beds, yet low occupancy of beds, includes low health budget from the government and financial, geographical and cultural barriers [3], and also the perception of low quality health services by the community [4]. As secondary or tertiary health care facilities, these hospitals receive medical cases referred by public health centers or medical practitioners from sub-district and community levels. The hospitals also receive directly coming inpatients and outpatients.

At sub-district and community levels, public health centers and their community network of village maternity units and integrated health posts are available providing basic services in maternal and child health, family planning, immunization and nutrition. A public health center is normally staffed by one physician and a number of paramedical workers, a village maternity unit by one village midwife, and a village integrated health post by community health cadres [5].

The Indonesian health workforce has been steadily increasing but the number of physicians, in particular medical specialists, is still relatively small. These health professionals work mostly in cities and towns [6]. The number of paramedical workers, in particular village midwives, has increased rapidly following the mass village midwife training and deployment program introduced in the early 1990s [7]. The issue concerning the midwife is not the number available but their quality and distribution [8-11]. A significant number of villages in many districts have reported not having a village located midwife available [9,12]. Many village midwives prefer to stay at the sub-district capital, not the village [13]. There is also reluctance to use the trained but young and often unmarried midwives by the village women [14] and there is also opposition from the traditional birth attendants (TBAs) [15].

As in many other developing countries, the contribution of private sector to the health care market is significant and growing [16-18]. Private providers are varied, and provide critical public health services. However, the large and growing private health providers, including the pharmacists and drug sellers, have not yet been optimally coordinated to address the public health problems [18].

The health reform, in particular through health decentralization, has been initiated since 2001 with the intention to bring better services closer to the community. In fact, the decentralization has not yet significantly improved the performance of the health system [19]. The structural problems make management of the health decentralized system as a whole difficult. Despite progress in health provision, health inequity continues. Health status and service access differs substantially between urban and rural areas [20] and in particular between the rich and the poor [21]. Following the economic crisis in 1998, the government intensified efforts to preserve and enhance access of the poor to quality health services through the social safety net program [22,23] and the use of health cards [24,25]. The fund for this program was provided to public health facilities, including hospitals and health centers [26,27] and after 2005, through social health insurance (*Jaminan Pelayanan Kesehatan Masyarakat *or Jamkesmas) [28,29]. This social health insurance scheme now covers 76.4 million people considered poor [30]. To further enhance access of the poor to health services, the government launched in 2007 the conditional cash transfer (CCT) program [31,32] and, by 2010, the program had covered 816,376 poor households (unpublished data from Ministry of Social Affair, 2011). To empower the poor communities economically and socially the government, through the National Team for Accelerating Poverty Reduction (TNP2K), initiated a community-based development program in 2007, called the National Community Empowerment Program (PNPM). This PNPM, as a continuation of the Sub-district Development Program introduced in 1998, has a target to cover 16 million poor people in all Indonesian villages by 2015 [33].

This paper has been prepared with two inter-related objectives: (1) to examine the levels and trends in socio-economic inequity in health in Indonesia; and (2) to identify the critical factors constraining the efforts to improve the health of the poor.

## Methods

The measures for community health status include child mortality, indicated by under-five child, infant and neonatal mortality rates, and child growth, indicated by the z-scores of weight for age. Measures on coverage of basic maternal and child health services were used to indicate access to health services. These health outcome measures are differentiated by household socio-economic and other relevant social variables to indicate health inequity. Households are ranked into socio-economic quintiles or deciles based on assets or expenditure depending on the data availability. The same household socio-economic measure is used in comparing the socio-economic inequity of health across times or regions [34].

The analysis uses an ecological perspective of population health determinants [35] to identify institutional and policy level factors considered critical in challenging the health program efficiency and effectiveness. Formal and informal systems, structures and norms, which may constrain or promote recommended behaviours, are considered as institutional factors, while national and local laws, policies and rules, which regulate or support healthy actions and practices are considered as health policy factors. These factors influence program efficiency and effectiveness through policy relevance and implementation [36].

Data for examining trends and levels of health inequity have been taken from two major sources: the Indonesia Demographic and Health Surveys (IDHS) from 1987 to 2007, and the National Socio-Economic Surveys (NSES) from 2000 to 2009. Quality of data from IDHS and NSES is considered adequate as the data are frequently used for population, health and economic analysis [37,38]. The IDHS has been conducted since 1987 for every three to five years, while the NSES since 1963 for every year. These surveys, conducted by the Central Board of Statistics, are nationally representative covering almost all the Indonesian geographic regions with a random sample of 26,000 to 41,000 households for each IDHS, and 190,000 to 292,000 households for each NSES. The IDHS collected demographic and health data through face-to-face interviews with reproductive aged women using structured questionnaires. The NSES 2000 to 2005 contained health modules which collected health data, including anthropometric measures of under five year old children.

Independent from the IDHS and NSES, data for explaining factors considered critical in affecting the health program efficiency and effectiveness were obtained through multiple means, including review of related published and unpublished research reports and government documents, and direct unstructured interviews with relevant actors in the government, the parliament, the donor, and the NGO communities. The relevant actors meant their working field is health related. The interviews, conducted during years 2009 to 2011 in the national capital and the West Java Province, did not intend to identify all critical institutional factors or to demonstrate conclusively their causal impact, but tried to uncover key factors, including their possible role in causal processes. Around 30 interviews were completed with each averaging 60 minutes in length. Topics addressed during interviews varied depending upon the interviewee's background, position, and interest, but the interview discussions centred on issues associated with health policy-making and programs. Not all interview results are presented, but those that are considered relevant for the explanation of constraining factors to improve the health of the poor. The analysis tried to provide descriptions and explanations on associated issues of health policy formulation and implementation.

## Results

### Trends in health status

**Figure 1:**
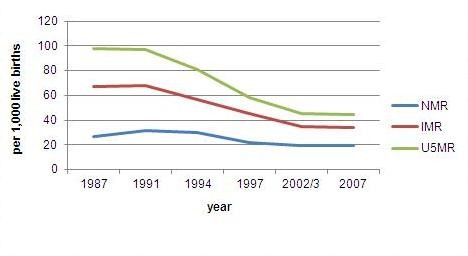
Trends of under-5, infant and neonatal mortality rates*, IDHS 1987-2002, Indonesia.

The health of Indonesians has generally improved during the past decades as indicated by improved survival of infants and children. Data from the Indonesia Demographic Health Surveys 1987 to 2007 show declining trends of under-5 and infant mortality rates, though the decline slowed down after 2002 (Figure 1). These mortality rates were computed from the information on date of birth, date of death, and date of survey for children born during last 5 years preceding the survey. Hence, the under five death is defined as the death below 5 years, the infant death is below 12 months, and the neonatal death is below 30 days. The child and infant mortality decline occurred in all social categories, in both the rich and the poor, and in both urban and rural populations. The mortality decline has been slower among the neonatal than among the post-natal ages. Most neonatal deaths are related to the prenatal period and delivery surrounding events, while the postneonatal deaths are more likely associated with risks related to environmental conditions after the delivery [39]. Compared to neonatal death causes, the risks related to environmental conditions are more responsive to available health technology advances, including better sanitation, the use of antibiotics and improved nutrition, that resulted in fewer infections, and, thus, the decline in postneonatal deaths [39]. Evidence suggests that reduction in neonatal mortality requires the introduction of expensive neonatal intensive care [40].

**Figure 2:**
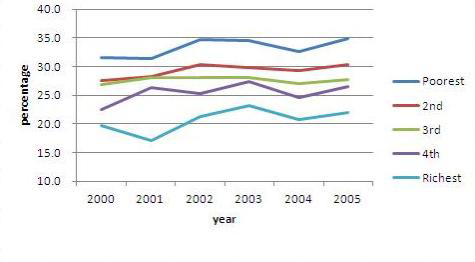
**Trends of underweight children by household expenditure quintile**.

Surprisingly, there is no sign of declining prevalence of undernourished children. The NSES showed a stable trend of underweight children at a relatively high prevalence of around 27% and the prevalence differed substantially by household socio-economic levels (Figure 2). A debate has recently emerged whether the child nutritional status in Indonesia has improved during the past decade as the National Basic Health Surveys (NBHS) conducted afterward by the Ministry of Health in 2007 and 2010 showed also a stable prevalence of underweight children, but much lower levels at around 18% [41].

The dramatic decline of underweight children that occurred within a relatively short time period from 27% in 2005 to 18% in 2007 would be unlikely. Rather than showing a decline, the above two different levels of underweight children would be more likely related to different methodologies between the two surveys in terms of the weighing scale used, method of weighing, type of personnel who measured the weight, and the child growth curve standard reference used. The NSES used 'Dacin' weighing scale to weigh the child of any age under five years, while the NBHS used 'AND' digital weighing scale to weigh the child aged 2 years and above, and used the same scale to weigh the child under two years of age but by weighing first the mother and then the mother and her child. The weight-for-age z-score is used to measure child's nutritional status. A child with a weight-for-age z-score of less than -2 SD is considered underweight. The NSES used the sub-district statistical officer to weigh the child, while the NBHS used trained university health students or new graduates to weigh the child. For measuring the weight-for-age z-score, the NSES used the National Center for Health Statistics/WHO international growth reference [42], while the NBHS used the World Health Organization (WHO) Child Growth Standards [43].

### Trends in health service access

**Figure 3:**
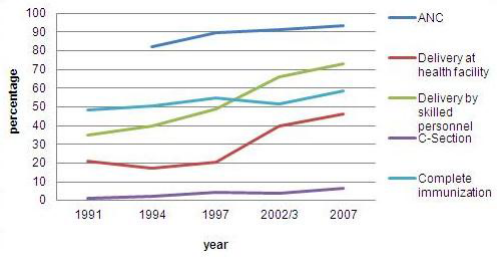
**Trends of antenatal care, birth delivery at health facility, by health personnel, and by C-section, and child completely immunized by first of age, IDHS, 1991-2007**.

The coverage of maternal health services has been steadily increasing in Indonesia. The percentage of pregnant women who had ever visited health personnel or a health facility for antenatal care was already high at 82% in 1994 and increased to 93% in 2007 (Figure 3). Compared to the percentage of pregnant women who conducted antenatal care, the percentage of birth deliveries attended by health personnel is much lower, and increased from 40% in 1994 to 73% in 2007 (Figure 3). These numbers indicate that there are still many women who received antenatal care but were not attended by health personnel during their birth delivery. Compared to the percentage of deliveries attended by health personnel, the percentage of deliveries that took place at a health facility is also much lower, and increased from 18% in 1994 to 46% in 2007 (Figure 3). These data suggest that not all the deliveries attended by health personnel took place at a health facility. In Indonesia, more than half of birth deliveries took place at home. Significant proportions of deliveries in several regions are attended by non-health professional [44].

**Figure 4:**
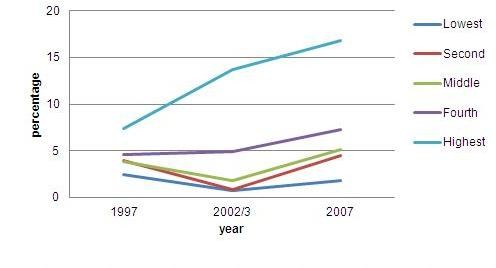
**Trends of birth deliveries with C- section by wealth quintile, Indonesia, 1997-2007**.

Evidence suggests that around 10% to 15% of pregnancies in any population would enter into some degree of maternal complications [45,46]. These complications, in particular hemorrhage, infections, or toxemia, which frequently occur at times around delivery, require emergency obstetric care. There is no precise rule regarding which maternal complications require what kind of emergency obstetric care, but between 5% and 10% of deliveries are expected to require caesarian-section (C-section) [46,47]. Given this knowledge, the percentage of deliveries with C-section of less than 5% might indicate unmet need of obstetric services, while more than 10% might indicate some abuse of such services. The Indonesian data show that the percentage of deliveries with C-sections has increased significantly from only one percent in 1991 to 7% in 2007 (Figure 3). As an indicator of accessing life saving services, the percentage of deliveries with C-section differs markedly between the rich and the poor (Figure 4).

On the other hand, the coverage of basic child services has not yet significantly improved. The percentage of children completely immunized by 12 months of age seems to persist at levels of 50% to 60%. These levels are far below the universal coverage of 100%.

### Socio-economic inequity of health

The data show persisting socio-economic inequity in health in terms of child mortality and child growth, in particular by mother's education, urban-rural residence and wealth status of the household. The ill health indicators are higher for children of mothers with lower education, residence in rural areas and for households with lower wealth status.

#### Infant mortality differentials

The infant mortality rate, which is often used to indicate the community health status and welfare, differs considerably by mother's education, urban-rural residence, and household wealth status (Table 1). Since mother's education and household wealth status are closely correlated, these two variables might confound each other in affecting infant mortality. The data show that the higher the mother's education the higher the proportion of richest households (Table 2), the higher the household health expenditure (Table 3). Nevertheless, the statistical analysis shows that both the mother's education and the household wealth status have their own independent effect on health outcomes. Even after controlling for potential confounding variables, the mother's education and the household wealth status retain their own effect on use of skilled birth attendant for delivery, completed immunization by first 12 months of age, and underweight children (Table 4).

**Table 1:** Trends and differentials of infant mortality rate, Indonesia, 1987-2007

	Survey					
	
	1987	1991	1994	1997	2002/3	2007
Total	75.2	74.2	66.4	52.2	43.0	39.0
Mother education						
No education	98.8	89.0	90.5	77.5	67.1	72.9
Primary	71.3*	81.1	70.4	58.8	50.8	46.3
Secondary/higher	33.9	34.6	39.5	28.0	29.0	28.8
Region						
Java-Bali	70.3	78.8	66.5	46.8	39.8	33.5
Non Java-Bali I	83.7	69.2	66.8	58.3	46.6	46.2
Non Java-Bali II	75.5	65.9	65.3	60.7	49.0	44.6
Residence						
Urban	50.9	57.2	43.1	35.7	31.9	30.6
Rural	84.1	81.0	75.2	58.0	52.4	44.8
Wealth quintile**						
Lowest	-	-	-	78.1	60.6	55.8
Second	-	-	-	57.3	50.3	47.3
Middle	-	-	-	51.4	44.0	32.5
Fourth	-	-	-	39.4	36.4	28.8
Highest	-	-	-	23.3	17.1	26.0

Source: Reports of the Indonesia Demographic and Health Surveys (IDHS) 1987 to 2007 Infant mortality rates were directly estimated from dates of birth, death, and survey of births born during the last 10 years preceding the survey

*Averages of IMR between two mother education categories: some primary (82.5) and primary completed (60.1)

**Based on household assets.

**Table 2:** Mother's education by household income quintile, Indonesia, 2009

	Income quintile				
Mother's education	Poorest	2nd	3rd	**4**^**th**^	Richest
No school	42.9	26.4	16.7	10.0	4.0
Elementary school	35.8	26.7	19.3	13.0	5.2
Junior high school	25.6	23.9	21.9	18.8	9.8
Senior High School	14.2	17.7	21.3	24.4	22.4
Academic	3.8	8.1	15.1	27.4	45.6
University	2.3	5.3	10.2	24.3	57.9

Source: Figures were computed from the data of National Socio Economic Survey (NSES), 2009

**Table 3:** Mother's education and annual household expenditure (rupiah), Indonesia, 2009

Mother's education	Household expenditure	Household health expenditure	% health expenditure
No school	16,300,000	334,535	2.1%
Elementary school	17,800,000	399,022	2.2%
Junior high school	20,500,000	558,002	2.7%
Senior High School	26,400,000	826,785	3.1%
Academic	36,800,000	1,357,580	3.7%
University	46,000,000	1,619,476	3.5%

Source: Figures were computed from the data of National Socio Economic Survey (NSES), 2009

**Table 4:** Logistic analysis of skilled birth attendance, completed immunization, and underweight children

	Skilled birth attendance*		Completed immunization**		Underweight***	
	
	Exp(b)	CI	Exp(b)	CI	Exp(b)	CI
**Insurance**						
No insurance	1.00		1.00		1.00	
Social health insurance	0.97	0.97-0.97	1.08	1.08-1.08	1.08	1.08-1.08
Others insurance	1.59	1.58-1.60	1.14	1.14-1.15	0.90	0.90-0.90
**Decile expenditure**						
Decile 1	1.00		1.00		1.00	
Decile 2	1.24	1.23-1.24	1.19	1.18-1.19	0.96	0.96-0.97
Decile 3	1.37	1.37-1.38	1.15	1.15-1.16	0.90	0.89-0.90
Decile 4	1.42	1.41-1.42	1.17	1.17-1.18	0.89	0.89-0.90
Decile 5	1.53	1.52-1.54	1.22	1.22-1.23	0.83	0.82-0.83
Decile 6	1.61	1.60-1.62	1.29	1.28-1.29	0.90	0.90-0.90
Decile 7	1.72	1.72-1.73	1.30	1.30-1.31	0.82	0.82-0.83
Decile 8	1.86	1.85-1.87	1.33	1.33-1.34	0.77	0.77-0.77
Decile 9	2.21	2.20-2.23	1.29	1.28-1.29	0.74	0.74-0.75
Decile 10	3.42	3.38-3.45	1.48	1.47-1.48	0.60	0.60-0.61
**Father's education**						
No school	1.00		1.00		1.00	
Primary school	1.14	1.14-1.15	1.09	1.09-1.10	0.97	0.96-0.97
Junior high school	1.64	1.63-1.65	1.15	1.15-1.16	0.95	0.95-0.96
High school	2.28	2.27-2.29	1.18	1.18-1.19	0.91	0.91-0.91
Academy	2.11	2.08-2.14	1.17	1.16-1.17	0.80	0.80-0.81
University	3.63	3.59-3.68	1.18	1.18-1.19	0.94	0.93-0.04
**Mother's education**						
No school	1.00		1.00		1.00	
Primary school	1.37	1.35-1.37	1.16	1.16-1.17	0.89	0.88-0.89
Junior high school	2.37	2.36-2.38	1.28	1.28-1.28	0.84	0.84-0.85
High school	3.61	3.60-3.63	1.32	1.32-1.33	0.79	0.79-0.79
Academy	7.28	7.17-7.40	1.39	1.38-1.39	0.65	0.64-0.65
University	7.99	7.85-8.12	1.27	1.26-1.27	0.55	0.55-0.56
**Rural urban residence**						
Rural	1.00		1.00		1.00	
Urban	0.43	0.42-0.43	0.86	0.85-0.86	1.00	0.99-1.00
**Region**						
Java-Bali	1.00		1.00		1.00	
Sumatera	1.36	1.35-1.36	0.74	0.74-0.74	1.25	1.25-1.25
Kalimantan	0.56	0.56-0.57	0.82	0.82-0.82	1.50	1.49-1.51
Sulawesi	0.51	0.51-0.51	1.14	1.13-1.14	1.48	1.47-1.48
Maluku-NTT-NTB	0.40	0.40-0.40	1.06	1.06-1.06	1.73	1.72-1.74
Papua	0.31	0.30-0.31	0.60	0.60-0.61	1.26	1.25-1.28

*Source: Computed from the data of National Socio Economic Survey (NSES), 2009

**Source: Computed from the data of National Socio Economic Survey (NSES), 2009; among children less than 5 years of age

***Source: Computed from the data of National Socio Economic Survey (NSES), 2005

The data show consistent socio-economic differentials in infant mortality during the past two decades (Table 1). Infant mortality rates are around two and half times lower for mothers with secondary or higher education than for mothers with no education, and around two to three times lower for households with the lowest wealth quintile than for households with the highest wealth quintile. Levels of infant mortality are lower for Java-Bali as compared to the outside Java-Bali regions, especially during the past decade (Table 1). Consistently, the 2005 NSES data show that Java-Bali, as compared to the other islands, has the lowest proportion of underweight children (Table 4). This observation is in line with the fact that Western Indonesia, especially Java and Bali, is significantly more advanced than the Eastern Indonesia in terms of infrastructure and socio-economic development [20].

#### Socio-economic differentials of child nutritional status

The different trends and differentials between child mortality and child growth measures suggest that the two types of indicators should be used together to indicate the community health status. The data show no clear differential in infant mortality between Java-Bali and the outside Java-Bali regions (Table 1), but the child growth indicators are clearly lower for the regions outside Java-Bali, including Sumatera, Kalimantan, Sulawesi, Maluku, West Nusa Tenggara, East Nusa Tenggara and Papua. Even after controlling for the socio-economic variables, the proportions of underweight children less than 5 years of age were 1.3 to 1.7 time higher for the regions outside Java-Bali than for the Java-Bali regions (Table 4). Also, the proportions of deliveries attended by health personnel, which measures health service access, were two to three times lower for the regions outside Java-Bali, except Sumatera (Table 4).

The data indicate persisting differentials of child nutritional status, indicated by the proportion of underweight children by mother's education and household expenditure (Figure 2 and Table 5). The proportion of underweight children less than five years of age was almost double for children with mothers with no schooling compared to those with mothers with an academic/university education. Almost twice the proportion of children under five from the poorest quintile households are underweight compared to those from the richest quintile households.

**Table 5:** Percentage of underweight children*, Indonesia, 2000 to 2005

	Year					
	
	2000	2001	2002	2003	2004	2005
Total	24.6	26.1	27.3	27.5	28.2	28.0
Mother's Education						
No school	28.9	30.7	32.3	33.7	32.7	33.4
Primary school	25.8	27.7	28.8	29.9	31.1	30.0
Junior high school	23.3	24.8	26.6	27.4	25.6	27.5
High school	20.7	22.3	23.0	24.1	22.0	24.0
> High	16.3	14.9	20.2	19.4	17.0	18.7
Expenditure Quintile**						
Lowest	28.3	28.9	32.1	33.1	30.7	32.8
Second	24.8	27.5	28.1	28.8	29.3	28.6
Middle	24.4	26.7	25.7	27.0	27.1	26.8
Fourth	21.2	24.2	23.5	25.4	23.7	25.3
Highest	18.1	18.3	19.5	21.1	20.3	20.3

Source: Figures were computed from the data of National Socio Economic Surveys (NSES) 2000 to 2005

*For children under five years of age; based on the National Center for Health Statistics/WHO international growth reference [42]; categorized as underweight if the weight-for-age Z-score is less than -2SD.

**Based on household expenditure.

Logistic regression analysis, using the 2005 NSES data, was performed to examine the net effect of demographic and socio-economic variables on child nutritional status, measured through the proportion of underweight children. Household expenditure, mother's education and residence in the Java-Bali region rather than other regions stand out as strong predictors for child nutritional status. The proportions of underweight children are higher for lower household expenditure, lower mother's education, and for residence in the regions outside Java and Bali.

The mother's education, as compared to the father's education, has a much stronger effect on child nutritional status. The risk of having underweight children is almost double for mothers with no schooling than for mothers with an academic or university education, but is almost the same between different levels of father's education (Table 4). Using data from the NSES 1992 to 1999, the previous study also demonstrated the very strong protective effect of mother's education on child nutritional status [48]. The role of mother's education in improving child health has been widely recognized in the literature [49]. Maternal education improves child health through a number of ways. Maternal education improves child nutritional status by increasing mothers' decision-making power in allocating family resources that promote their child nutrition and health [50-52]. One study in Bolivia suggested that socio-economic factors are the most important pathways linking maternal education and child nutritional status [53].

Surprisingly, residence in urban or rural areas, after controlling for the other socio-economic variables, shows no effect on child nutritional status. Hence, one might conclude that the commonly noted urban-rural differentials of health status and service access can be explained by the urban-rural differentials in mother's education and wealth status [20]. Rural areas are known to have less availability of, and access to health services, which could be the other factors in the rural-urban differential in child mortality and child nutrition, but it should be stressed here that mother's education and household wealth can overcome this disadvantage of rural areas.

#### Health service access differentials

Health service access has improved, but the rich-poor gap in health service access remains. The percentage of birth deliveries attended by health personnel increased from 49% in 1997 to 73% in 2007, while the percentage of birth deliveries took place at health facility was about half lower, but also increased from 21% in 1997 to 46% in 2007 (Table 6). These figures suggested about half of birth deliveries attended by health personnel were taking place at home. Percentages of birth deliveries attended by health personnel and took place at health facility differed considerably by household wealth status. The 2007 IDHS data show the percentage of birth deliveries attended by health personnel was 96% for the highest and only 44% for the lowest wealth quintile of the population, while the percentage of birth deliveries took place at health facility was 83% for the highest and only 14% for the lowest wealth quintile of the population (Table 6).

**Table 6:** Birth deliveries by health personnel and at health facility by wealth quintile, Indonesia, 1997-2007

	By health personnel			At health facility		
	**1997**	**2002/3**	**2007**	**1997**	**2002/3**	**2007**

Total	49.1	66.3	73.0	20.7	39.8	46.0
Wealth quintile*						
Lowest	21.3	39.8	43.8	3.9	10.8	13.6
Second	34.9	56.0	66.3	8.4	24.7	31.7
Middle	48.1	68.7	78.8	17.8	37.9	47.9
Fourth	64.5	80.6	87.2	29.8	53.4	61.7
Highest	89.2	93.6	95.5	52.6	81.3	83.3

Source: Reports of the Indonesia Demographic and Health Survey (IDHS), 1997 to 2007 *Based on household assets.

Information on the percentage of deliveries by Caesarian Section is useful to show the extent of unmet need for emergency obstetric services. This indicator is particularly sensitive to socio-economic inequity given the relatively high cost of C-section deliveries in Indonesia [54,32]. Over the ten years from 1997 to 2007, the percentage of deliveries by C-section for the lowest wealth quintile population remained far below 5%, but for the highest wealth quintile population it had increased sharply to reach over 15% (Figure 4). Thus, the rich-poor gap in access to potentially life-saving emergency obstetric care widened [55].

Similar to the access to basic maternal health services, access to basic child services also differs considerably by wealth status. Data from the 2007 IDHS showed that the percentage of children completely immunized by 12 months of age is only 39% for the lowest, but 75% for the highest wealth quintile of the population (Table 7).

**Table 7:** Percentages of children completely immunized by 12 months of age, Indonesia, 1997-2007

	1997	2002/3	2007
Total	51.4	58.6	48.3
Wealth quintile*			
Lowest	42.9	37.1	39.4
Second	47.2	46.6	53.0
Middle	46.5	52.5	58.1
Fourth	58.0	58.1	68.0
Highest	72.1	64.7	74.9

Source: Reports of the Indonesia Demographic and Health Survey (IDHS), 1997 to 2007 *Based on household assets.

The above rich-poor gap means the cost, particularly for the Indonesian poor, is a constraint for accessing health services. From the demand side, health care utilization requires cost for service fee and other expenses, including medicines, transportation, food and drink, and other related costs during health care visitation. The government subsidizes health care financing through public sector but the subsidy has not yet been adequate to make health care services free, even for the poor. Even the public health care facility in reality applies a service fee [26]. In Indonesia, the health care services are provided through a mixture of public and private sectors. The role of the private sector in health care service provision is significant and increasing [26,18,17]. The contribution of the private sector to the health care expenditure is estimated about 70% to 75% [26]. The private sector health care financing comes from out of pocket payments from individuals and households, reimbursement by corporations, third party payments through private insurance companies, and direct health services provision by large firms.

### Factors constraining efforts to improve the health of the poor

To be effective, efforts to improve the health of the poor should translate into routine quality services that can reach the majority of the poor [56,57]. While meeting the community needs, the policies should be commonly perceived, accepted and implemented by relevant service providers [54,58]. The Indonesian government has developed priorities for addressing the health of the poor, but in reality some factors at the institutional and policy levels have constraints which challenge the efficiency and effectiveness of social and health programs.

#### Institutional level: the high cost system of electing formal governance leaders

The high cost system of electing local governance leaders, which emerged after the adoption of the regional autonomy in early 2000s, has become an important factor at the institutional level, constraining efficiency and effectiveness of social and health programs. Under the regional autonomy, the local governance leaders, i.e. the Governor (*Gubernur*) and the Deputy Governor (*Wakil Gubernur*) at the provincial level, the Regent (*Bupati*) and the Deputy Regent (*Wakil Bupati*) at the district level, and the Mayor (*Wali Kota*) and the Deputy Mayor (*Wakil Wali Kota*) at the city level, were locally elected every five-year by the community. At present, there are 33 provinces and 497 districts, including cities, in Indonesia [59].

One should spend billions of *rupiah *to become a local leader candidate. The money is used to gain support from political parties with the parliamentary majority and to attract sympathy from community members. In many cases, a leader was elected not because of his/her leadership and professionalism, but money. Since salary of a local leader for a five-year period of appointment will not meet the money he/she already invested, once elected the local leader would first think how his/her invested money could grow or at least be returned. One recent study indicated that leaders elected in this way must recuperate the costs, leading to corruption, poor governance, and sub-optimal leadership [60]. Below is a quote from one national newspaper on the incompatibility of the high cost system of electing formal local leaders to the fight against corruption.

*"The government has spent almost 4 trillion rupiah to implement general election of local leaders in the year of 2010. In that year, there will be 244 elections of local leaders: 7 provinces, 202 districts, and 35 cities. In addition, a candidate should spend around 5 billion rupiah (USD555,556) at the district level and around 20 billion rupiah (USD2,222,222) at the province level. This is a paradox when these leaders are supposed to fight against corruption, said an economist from the Gajah Mada University." *[61]

Under the regional autonomy, the local governance leaders, in particular *Bupati *and *Wali Kota *at the district level, have the power to decide, fund and control the development programs in their locality [19]. In reality, however, these leaders would give priorities to development programs considered profitable to them. Social and health development programs, which are mostly not profitable to the leaders, would not receive real commitments. One recent study showed that the implementation of the decentralization regulation decreases the responsiveness of the directly elected leader to the health and infrastructure sectors [62]. The decrease in the responsiveness was even more when these leaders were supported by political parties holding the parliamentary majority [62]. The threats of the high cost local leader election system to health are especially higher in poor cities or districts due to the lack of understanding and awareness of new leadership of local governments towards the benefits of and funding for public health services [21]. Presented below are quotes of interview with one health economist and one national NGO activist commenting on decentralization and health.

*"Today, many local leaders (District/City Head) and parliament members in more than 400 districts/cities, who were previously inexperienced bureaucratic, amateur politicians, and businessmen, are not well informed about health. Their work priorities are mostly on physical development and investment." (A national health economist)*.

*"Now health might be less important to local government, because there are more tangible programs, notably physical infrastructure .... It is a big challenge for those who care about peoples' health and welfare ... " (A national health NGO activist)*.

Under the above circumstances, corruption, defined as 'misuse or abuse of entrusted power for private gain [63,64], is rampant [60], and it is very difficult to expect proper public health services unless there is some degree of moral obligation or motivation [21]. In the health sector, corruption might relate to the roles and relationships among different players including regulators, payers, providers, consumers and suppliers interacting in complex ways [64]. Public officials are considered corrupted when they use their position and power not to benefit the public good, but instead benefit themselves and others close to them [64]. Hence, corruption is a critical factor affecting negatively the efficiency and effectiveness of development programs, including health programs.

#### Policy level: confused leadership roles in the health sector

Despite regional autonomy, many relevant informants in this study still expressed high expectations of the critical leading role of the Ministry of Health (MOH) in health policy-making and in its implementation. For example, one informant from a non-governmental organization expressed his view that the MOH should take more initiative in addressing the health issue.

*"Everything depends on the MOH. I do not expect the president to talk about Safe Motherhood. Even when the president is a woman or a doctor, I do not expect he/she will talk about it. The president has many things to think of, am I right? Economic matters, national defence matters, etc. But the MOH should be the one that pushes the president to address this matter." (A national health NGO activist)*.

In line with the above view, another informant said the MOH should play an enhanced co-ordination role in addressing the health issue.

*"Sometimes people put the blame on either the system or human resources. People always say that the health issue must be understood and implemented by other sectors. Who has to change this behaviour? It should be the Ministry of Health. In Indonesia, there isn't any coordination... " (A national politician with the health background)*.

Two different informants, one from the non-health but related sector and the other from a professional organization, commented on the confused roles and functions within the MOH that might translate into the lack of health program coordination.

*" ... Every 1 or 2 month we meet. We have this reproductive health forum, held by the Ministry of Health. But there are too many participants, so we cannot find a common strategy. People go to the meeting and there is a lot of discussion and the leaders also always leave. ... They do not coordinate the meeting, they just participate. They do not influence much ... just attend the meeting." (A senior personnel from the health related sector)*.

*"Maybe the root cause is at the MOH. Why? Their directorates do not integrate. Have you not been invited to different workshops organised by different directorates within the MOH, but on similar topics?" (An expert from the health professional organization)*.

The lack of co-ordination within the MOH is compounded by a perception at the central level that the devolution of power to district levels has too greatly reduced the MOH's ability to co-ordinate because of a lack of incentive for the districts to take heed of the MOH's direction. A senior official from the MOH said:

*"It's because of the decentralisation they (District and municipality staff) don't want to come when I invite them to the meeting. When I give them the information, they do not listen ..." (A senior health officer)*.

One review pointed little coordination between district health officials and central public health programs, or between the district officials and private providers [18]. Different perceptions of health policies by different sectors and management levels have also disintegrated their complementary roles and responsibilities [65].

#### Policy level: equity is not yet a key indicator for health program achievement

Health inequity is not a new issue, but remains relevant to be addressed in the context of current health development program. Increasing concern on health inequity in Indonesia emerged when new legislation on regional autonomy was enacted in 2002 (Act no 22/2002). Many policy makers thought that regional autonomy might widen the existing health inequity, and promote unfavorable regional and urban-rural distribution of health personnel [21]. Nonetheless, to this day, health equity has not been used as a key indicator for measuring the achievement of the health program. Instead, health programs at the national and local levels generally use service coverage indicators as measures of the program achievements. One review has indicated that equity-oriented public policies and equity-sensitive program monitoring could overcome inequity problems [66].

#### Policy implementation level: the weak health system capacity

To be effective, the health system should have the capacity to perform successfully three public health core functions: assessment, policy development, and assurance [67]. Such a capacity requires adequate information, organizational, physical, human, and fiscal resources [68]. In Indonesia, the lack of these resources contributes to the weak health system capacity. Information required for health development planning, implementation and monitoring, in particular at local levels, is limited. The practical guidelines of health program implementation are lacking, and if available, they are not properly distributed and used at services levels.

The health policy is not commonly perceived and understood by relevant stakeholders, and assurance for quality health program implementation is lacking. Below is a quote from an interview with a national health activist on the lack of health quality assurance.

*"The government stated they have a policy to reduce maternal mortality but the maternal mortality remains high. What extent does the MOH react? The policy of the government [MOH] has not been clear. What programs? What objectives? Most health programs tended to be a political move, not adequately technically supported ..., for example, doctors to be deployed were not briefed on their objectives and tasks but administrative matters and the country ideology. The briefing often has nothing to do with the program." (A national health activist)*.

Budget allocated to the health program is often not adequate [69]. The health budget, particularly at the local levels is commonly planned with a limit for the maximum amount which has not changed much from year to year, even after regional autonomy began. An analysis on health program budgeting has identified several issues: 1) low overall budget, 2) low budget allocated for preventive measures, 3) budget for services inadequate, 4) late access to budgets, 5) not performance related, 6) fragmented, and 7) inefficient [70,42]. Of these issues, the late access to budgets each year is considered an important factor challenging the health program performance.

*"Before decentralization, around 35% of the national budget went to districts through various channels, but most came late so that their use and spending were disturbed. This late timing of budget realization had a negative impact on performance. SK Mendagri 29/2002 instructed an integrated budgeting plan in performance budgeting system. Nevertheless, this new integrated budgeting plan is still in a process not easily translated. Until today, late timing budget realization still commonly happens and disturbs the spending plan and health program performance......... The design of most projects is complicated; often not meeting the local situation needs, and is difficult to manage. Absorption of funds was generally bad. The budget spending was usually an upside down pyramid. There are too many donors, but lacked coordination. Often, the projects bought high tech equipment, but lacked utilization and maintenance. State policies are often not in line with the reality" (A national health economist)*.

Density of health care providers including doctors, nurses and midwives was low by international standards and varied widely by district. A study of 15 districts in Java showed none comes close to reaching the WHO cut-off of 2.5 health care providers per 1,000 population - in fact, 11 out of 15 districts had provider density below 1.0 [17]. Besides the low density, the unequal distribution of health providers, especially the professionals, is a major issue in the provision of quality of care, especially in rural areas. The unbalanced distribution of human health resources is associated with the differences in fiscal capacity of local governments to finance public health services and to hire public health professionals [21].

A study of midwifery provision in two districts showed 10% of villages do not have a midwife but a nurse as a midwifery provider; there is a deficit in midwife density in remote villages compared with urban areas [9]. The small number of obstetricians in rural areas and in areas outside Java and Bali islands has made emergency obstetric services a major concern [71]. Most medical specialists are concentrated in large provincial cities where top referral hospitals are located.

Studies also identified the health providers' lack of competence. One review indicated that many midwives struggle with their limited skills in the clinical procedures, as well as skills in interpersonal communication and management and supervision [71]. The midwives are often further hampered by inadequate transportation and - early in their appointment - deep distrust on the part of community members with regard to their skills, age and social abilities. The midwives are also often not equipped with adequate drugs. An assessment report in one district of West Java indicated that most of the 20 midwives interviewed did not have a supply of magnesium sulphate (a cathartic) as recommended by the Ministry of Health [72]. The midwives assigned to remote areas are less experienced; and these midwives manage fewer births which may compromise their capacity to maintain professional skills [9].

Health care facilities suffer from incomplete supplies of essential drugs and equipment. Several studies of health centers showed poor availability of the minimum necessary equipment and drugs [73,71]. These studies also identified various deficiencies in the provision of care, such as not washing hands before or after examining a patient, frequent reuse of non-sterilized needles, overuse of drugs and injections; 'flexible' personal interpretation of working hours; inadequate biomedical knowledge; arbitrary increases of fees; and emphasis of quantitative targets for service provision. The district hospitals often have chronic shortages of trained staff and essential supplies. One study in five districts of West Java showed not all the district hospitals have one obstetrician-gynaecologist available for 24 hours as intended [71].

#### Policy implementation level: lack of coverage and leakage to the non-poor

The social protection programs often miss many of the targeted poor, and instead provide fund to those that are not poor. The MOH reported that by 2010 the social health insurance covered 76.4 million people who were considered poor [30]. This number is about one-third of the Indonesian population. If the poor is defined as those within three deciles of household expenditure, the data of NSES 2004 to 2009 showed only around 50% of those who were receiving social insurance were really poor (Table 8). A half of the social health insurance went to the non-poor. Around 10% of social health insurance targeted for the poor went to the 30% richest, or the top three deciles of household expenditure. Studies on the Indonesia social safety net program also indicated that a large number of the poor were simply not covered by the program, and there was substantial benefit leakage to the non-poor [22,23].

**Table 8:** Coverage of Social Health Insurance (Jamkesmas) by household expenditure deciles

Year	Expenditure category			
	
	Bottom three deciles	Middle four deciles	Top three deciles	
2004	48.1	38.7	13.2	
2005	51.0	38.8	10.3	
2006	49.9	38.9	11.2	
2007	50.1	39.0	10.9	
2009	48.4	39.7	11.9	

Source: Computed from the data of National Socio Economic Survey (NSES), 2004 to 2009 Expenditure data from the 2008 NSES are not included because of the quality problem.

In 2007, the government launched the conditional cash transfer (CCT) program, called the 'Family Hope Program', to help access of the poor to basic education and health services. The program provides conditional cash to the very poor households for paying for transportation and other related costs of using basic health and education services. These very poor households were supposed to also receive social health insurance and a basic education fellowship. Social health insurance would cover health service cost, while the education fellowship would cover education cost. For the CCT program to be effective, the program participants should also receive assistance from other social protection programs, such as social health insurance and the education fellowship. An evaluation of the CCT program indicated that among the program participants only 70% reported to also receive social health insurance, and only 20% also received the education fellowship [74]. However, the evaluation did not report the number of CCT program participants who received both the social health insurance and the education fellowship.

Except for social health insurance, the coverage of other social protection programs is too small. For example, the CCT program covers 816,376 poor household in 2010 and planned to reach 1,116,000 poor households by 2011 [75,44]. This number is far smaller than the number of Indonesian poor. One study on the public funding of health services among the poor indicated that social protection programs, including the social safety net programs and health cards for the poor, have helped reduce inequity of health service access, but may not be sufficiently generous to protect all who were considered vulnerable [76,45].

## Discussion and conclusion

The Indonesian child and infant mortality rates have declined during the past two decades, but the decline is less impressive if it is compared to the progress made by neighboring countries, such as Malaysia, Thailand and Sri Lanka [11]. The decline of child and infant mortality rates have slowed down since 2002. Trends in other health indicators are not really promising. The prevalence of undernourished children is still relatively high. There is no indication of a decline in the trend of the prevalence of undernourished children. Two different national surveys, the NSES conducted during the period 2000 - 2005 and the NBHS conducted during the period 2007 - 2010, presented different levels of underweight children but both showed stable prevalence of underweight children. Trends in the percentage of children completely immunized by 12 months of age during the past two decades appear to be stagnant at levels of 50% to 60%, far below the target of 100%. Access to antenatal care and health personnel for delivery care has been steadily increasing, but maternal mortality remains high at a level which is among the highest in East Asia [77]. The fact that service coverage has greatly increased but has not been followed by improved health status indicates an issue of health service quality [78].

It is a matter of concern that socio-economic inequity in health is continuing to persist. In Indonesia, education of mother and household wealth status stand as strong predictors of health status and health service access. This persisting socio-economic inequity in health means that the currently operated public and private mix of healthcare delivery system fails to reach the poor who are in the greatest need of health services. The health care delivery system has compromised quality and equity in several ways through the combination of three factors: (i) inadequate health budget; (ii) a weak regulatory environment for delivering social and health services; and (iii) lack of transparency in governance [79].

The differences in the health outcomes between the rich and the poor are avoidable [56,80], therefore health inequity can be substantially reduced. Addressing comprehensively the social and economic causes of health disparities may be the best approach for closing the rich-poor health gap in health [81]. For effectively addressing such inequity, public policies should be equity-oriented and equity-sensitive program monitoring should be used [66].

Theoretically, there are three factors that might explain why the efforts to reduce health inequity are not effective. The first factor is lack of coverage. The programs cannot reach the poor or the numbers of those reached are too small to be able to impact on health outcomes. The second factor is lack of quality. Even when reaching the majority of the poor, the programs lack quality cannot make an impact on health outcomes. The third factor is lack of sustainability. Hence, sustained efforts should continue to deliver changes in these three factors. These sustained efforts should be directed towards (i) improving the adequacy of the state and local health budget; (ii) strengthening the regulatory environment for delivering social and health services; and (iii) making governance more transparent.

This study has highlighted institutional, policy and system level factors considered to be critical in constraining the 'pro-poor' social and health program effectiveness. These critical factors include the high cost system of electing formal governance leaders, confused leadership roles in the health sector, lack of use of health inequity indicators, the weak capacity of the health system in planning and budgeting, limited coverage of the health care and health insurance programs and misdirecting the program to the non-poor. These factors should be taken into account when developing and improving the pro-poor program strategies.

In conclusion, despite the government's efforts to improve the health of the poor, the rich-poor gap in health status and service access continues. Factors at institutional and policy levels are critical in contributing to the lack of efficiency and effectiveness for health programs that address the poor.

## Competing interests

The authors declare that they have no competing interests.

## Authors' contributions

BU has participated in the design of the study, interpreted data analysis results and drafted the manuscript. PKS provided policy and qualitative data, contributed to writing and editing the paper. FRU helped in the statistical analysis and contributed to writing and editing the paper. All authors read and approved the final manuscript.
